# The Association Between Hearing Loss and Completion of Advance Care Planning Among Older Adults

**DOI:** 10.1093/geroni/igaa057.1065

**Published:** 2020-12-16

**Authors:** Junghee Han, Junghyun Park

**Affiliations:** 1 Calvin University, Grand Rapids, Michigan, United States; 2 New York University, New York City, New York, United States

## Abstract

Background: Ensuring access to quality end-of-life (EOL) care for all older adults is emerging public health concern. Hearing loss (HL) is the third most common chronic disease affecting older adults and a major impediment to access healthcare services. However, little is known about the impact of HL on advance care planning for older adults. Method: A sample of 1,862 older adults (≥65 years) was drawn from the National Health and Aging Trends Study (NHATS). HL was determined by self-report and advance care planning was measured by asking if an individual completed living wills or the Durable Power of Attorney for Health Care (DPAHC). Covariates included age, gender, race, marital status, education, religion, nativity, depression, region, facility status, regular doctor availability, Medicaid, hospitalization, cognition, perceived health status and a presence of chronic disease. Results: Descriptive statistics revealed that nearly 67% of older adults with HL completed the DPAHC, and the majority of them (71%) also had living wills. Multivariable logistic regression analyses showed that HL was significantly associated with completion of DPAHC and living wills, after controlling for a list of covariates (OR=0.50, p<0.05). Conclusions: The findings show HL is a significant predictor of completion of any type of advance directives. Facilitating effective communication in advance care planning for older adults with HL is needed. Healthcare provider should make health information accessible to them to get quality EOL care.

